# Endobronchial Recurrence of Renal Cell Carcinoma 12 Years After Nephrectomy: A Rare Case Treated With Microwave Coagulation Therapy

**DOI:** 10.7759/cureus.97695

**Published:** 2025-11-24

**Authors:** Hirofumi Nakano, Yukihiro Sugimoto, Ryota Aoki, Yu Tsukasa, Tetsurou Takeuti, Takayuki Yamamoto, Sosei Abe, Masanori Takayama

**Affiliations:** 1 Department of Respiratory Medicine, Fukuoka Seisyukai Hospital, Fukuoka, JPN; 2 Department of Infection Diseases and Respiratory Medicine, National Defence Medical College Hospital, Saitama, JPN; 3 Department of Thoracic Surgery, Fukuoka Seisyukai Hospital, Fukuoka, JPN

**Keywords:** endobronchial metastasis (em), microwave coagulation therapy, nephrectomy, renal carcinoma recurrence, renal cell carcinoma

## Abstract

Endobronchial metastasis (EM) from renal cell carcinoma (RCC) is a rare manifestation of intrathoracic spread. RCC is known for its potential to recur long after nephrectomy, and EM may mimic primary lung cancer, complicating diagnosis and management. A 69-year-old male patient with a history of nephrectomy for RCC presented with progressive dyspnea. Imaging revealed a right hilar mass, and bronchoscopy identified a pedunculated polypoid lesion in the right main bronchus. Microwave coagulation therapy was performed to excise the lesion and achieve hemostasis. Histopathological analysis confirmed metastatic RCC. The patient was referred for systemic therapy following bronchoscopic intervention. This case highlights the diagnostic challenge of differentiating EM from primary lung cancer, especially in patients with a smoking history and elevated tumor markers. Bronchoscopic microwave coagulation therapy provided a safe and effective method for both diagnosis and airway management. Clinicians should remain vigilant for late recurrence of RCC, even more than a decade after nephrectomy. Bronchoscopic microwave coagulation therapy may serve as a minimally invasive option for the diagnosis and treatment of endobronchial lesions.

## Introduction

Endobronchial metastasis (EM) from extrapulmonary solid tumors is a rare manifestation of intrathoracic spread, reported to occur in approximately 3.6-8.6% of metastatic malignancies [1]. Renal cell carcinoma (RCC) is known for its potential to recur long after nephrectomy and frequently metastasizes hematogenously to the lungs, with pulmonary involvement reported in 30-50% of cases [2]. Furthermore, RCC is considered one of the most common primary tumors associated with EM [3].

Clinical symptoms of EM include cough, dyspnea, and hemoptysis, although asymptomatic cases have also been reported [4,5]. In the present case, differentiation from hilar-type lung cancer was required due to the patient’s long smoking history, elevated tumor markers, and imaging findings. Bronchoscopic biopsy was initially planned to target viable areas of the lesion, but the tumor was identified as a pedunculated polypoid lesion arising from the membranous portion of the bronchus. Microwave coagulation therapy (MCT) was selected to achieve hemostasis and excision, allowing for sufficient tissue sampling and definitive histopathological diagnosis. We report a case of EM from RCC that was controlled for a relatively long period using MCT.

## Case presentation

A 69-year-old male patient presented with progressive dyspnea in March 2023. He had a history of left nephrectomy for RCC performed 12 years earlier, as well as comorbidities including hypertension, hyperuricemia, and chronic kidney disease. He was a current smoker with a 43-year history (10 cigarettes/day).

**Figure 1:**
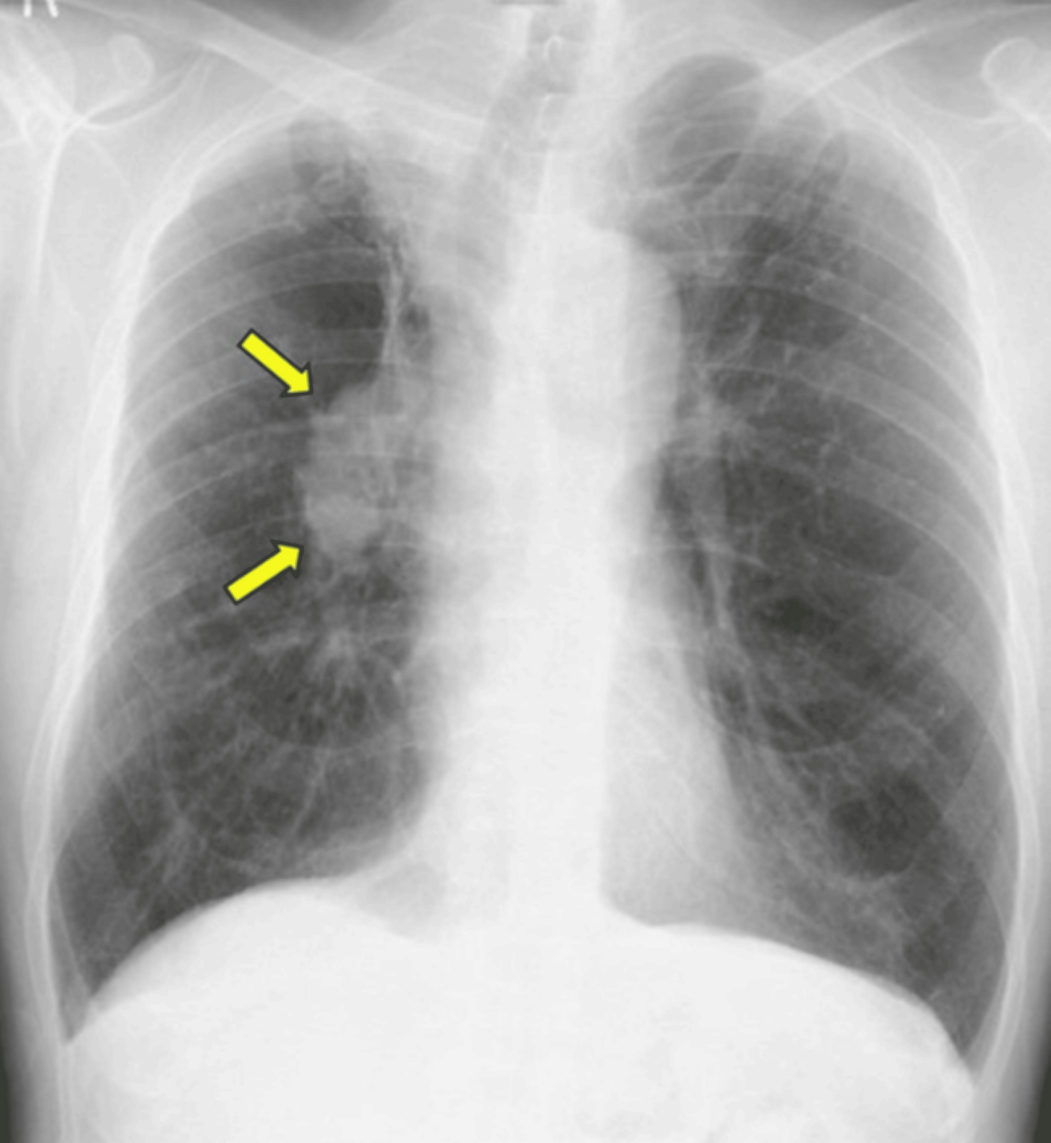
Chest radiograph showing a 60 × 35 mm mass (yellow arrows) in the right hilum

Initial evaluation at a local clinic led to a diagnosis of acute bronchitis and chronic obstructive pulmonary disease (COPD) exacerbation, but symptoms worsened, prompting emergency transfer to our hospital. On admission, vital signs were as follows: temperature, 36.3℃; blood pressure, 171/95 mmHg; pulse, 94 bpm; respiratory rate, 31 breaths/minute; and SpO₂, 99% (10 L/min via reservoir mask). Physical examination revealed bilateral wheezes without anemia, jaundice, or peripheral edema. Laboratory tests showed leukocytosis (WBC, 13,083/μL; neutrophils, 79.2%), mild elevation of C-reactive protein (CRP) and creatinine, and elevated CYFRA 5.2 ng/mL. Carcinoembryonic antigen (CEA) and pro-gastrin-releasing peptide (ProGRP) were within normal limits. Arterial blood gas analysis revealed pH of 7.435, PaO₂ of 105.3 mmHg, PaCO₂ of 31.0 mmHg, and HCO₃⁻ of 20.4 mmol/L (Table 1). Chest radiography revealed a 60 × 35 mm mass in the right hilum (Figure 1).

**Table 1:** Laboratoy tests WBC: white blood cell; RBC: red blood cell; Hb, hemoglobin; Ht: hematocrit; Plt: platelet; TP: total protein; Alb: albumin; AST: aspartate aminotransferase; ALT: alanine aminotransferase; LDH: lactate dehydrogenase; γ-GTP: gamma-glutamyl transpeptidase; BUN: blood urea nitrogen; Cre: creatinine; Na: sodium; K: potassium; Cl: chloride

Parameters	Result	Reference range
Complete blood count		
WBC	13083/μl	3000-9000/μl
RBC	528 × 10^4^/μl	430-570 × 10^4^/μl
Hb	15.5 g/dl	13.5-17.5g/dl
Ht	46%	40.8-49.6%
Plt	38.0 × 10^4^/μl	12.0-35.0 × 10^4^/μl
Biochemistry		
TP	7.7 g/dl	6.7-8.3 g/dl
Alb	4 g/dl	4.0-5.0 g/dl
T-bil	0.59 mg/dl	0.30-1.20 mg/dl
AST	25 U/l	13-33 U/l
ALT	29 U/l	6-30 U/l
LDH	181 U/l	119-229 U/l
γ‐GTP	42 U/l	10-47 U/L
BUN	21.3 mg/dl	8.0-22.0 mg/dl
Cre	1.2 mg/dl	0.60-1.10 mg/dl
Na	140 mEq/l	138-146 mEq/L
K	4.7 mEq/l	3.6-4.9 mEq/L
Cl	105 mEq/l	99-109 mEq/L
Serology		
CRP	0.52 mg/dl	0.00-0.30 mg/dl
CEA	3.7 ng/ml	0.00-5.0 ng/dl
CYFRA	5.2 ng/ml	≦3.5 ng/dl
ProGRP	33.5 pg/ml	≦81 pg/mL
Coagulation		
PT	75.80%	70.0-100.0%
APTT	30.7 sec	27.0-38.0sec

**Figure 2:**
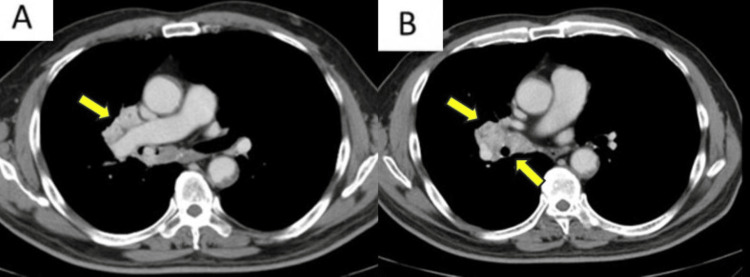
(Panel A) contrast-enhanced chest computed tomography revealing a heterogeneous tumor (yellow arrows) extending from the tracheal bifurcation to the right hilum, and (Panel B) causing stenosis of the right main bronchus

CT imaging showed a heterogeneous enhancing tumor extending from the tracheal bifurcation to the right hilum, with a 20 mm nodular lesion causing stenosis of the right main bronchus and enlarged mediastinal lymph nodes (#7 and right #10) (Figure 2).

**Figure 3:**
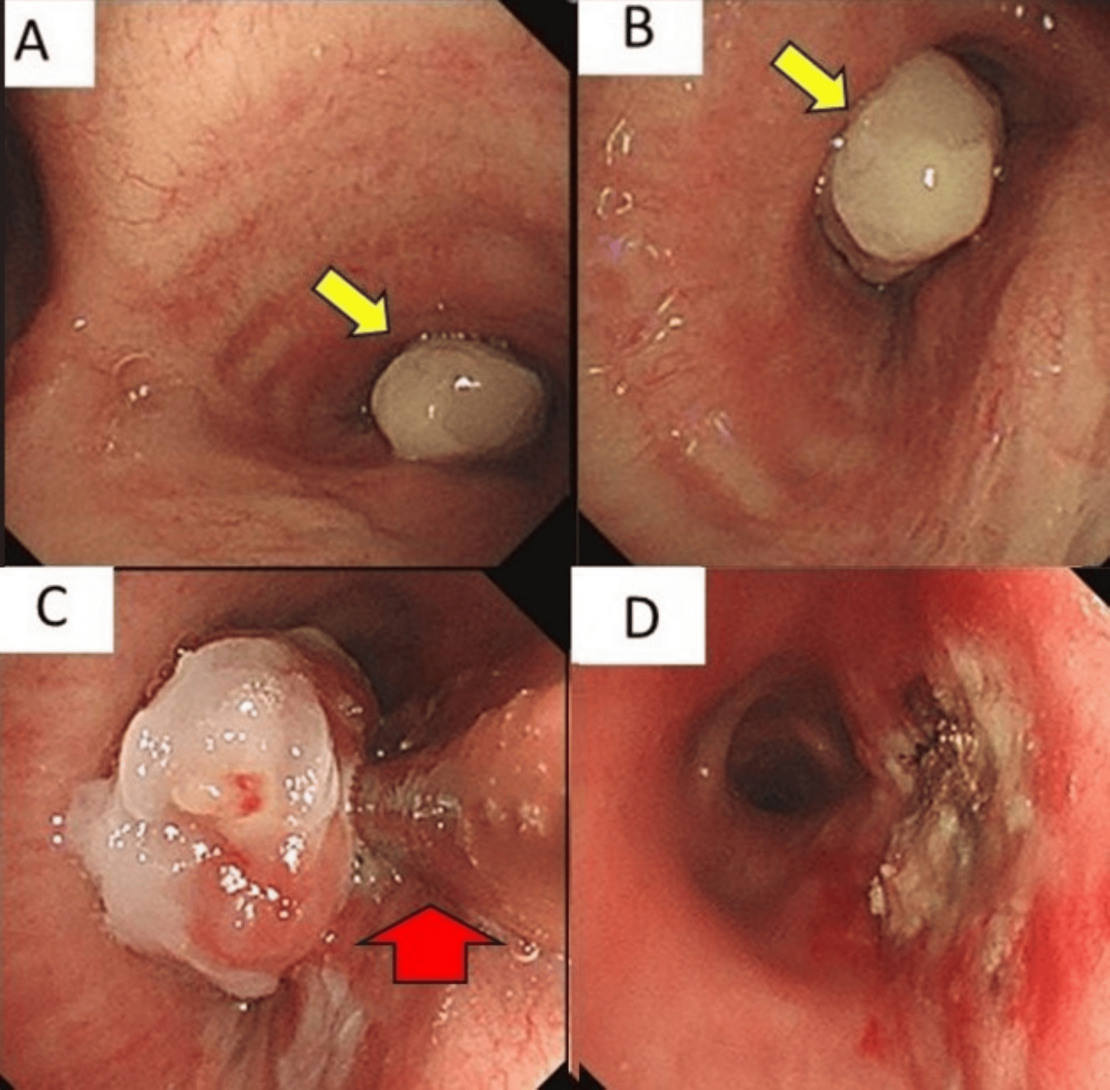
Bronchoscopic findings showing a pedunculated tumor (yellow arrows) protruding from the membranous portion of the right main bronchus (Panels A and B). The tumor was excised using a microwave coagulation device (red arrows) (Panels C and D)

No evidence of recurrence to lymph nodes or other organs was found on abdominal CT. Bronchoscopy revealed a pedunculated polypoid tumor protruding from the membranous portion of the right main bronchus, covered with necrotic material. Using a microwave coagulation device (Microtaze® AZM-550, Alfresa Pharma), the stalk was coagulated and excised with minimal bleeding. The 20 mm lesion was retrieved successfully, and the peripheral airway remained patent (Figure 3).

**Figure 4:**
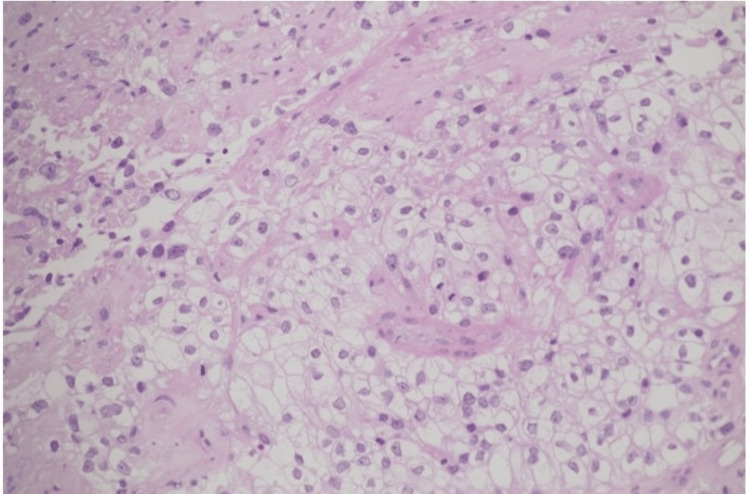
Histopathological examination with hematoxylin and eosin staining showing features consistent with clear cell carcinoma

Histopathological examination revealed atypical cells with clear cytoplasm (Figure 4).

**Figure 5:**
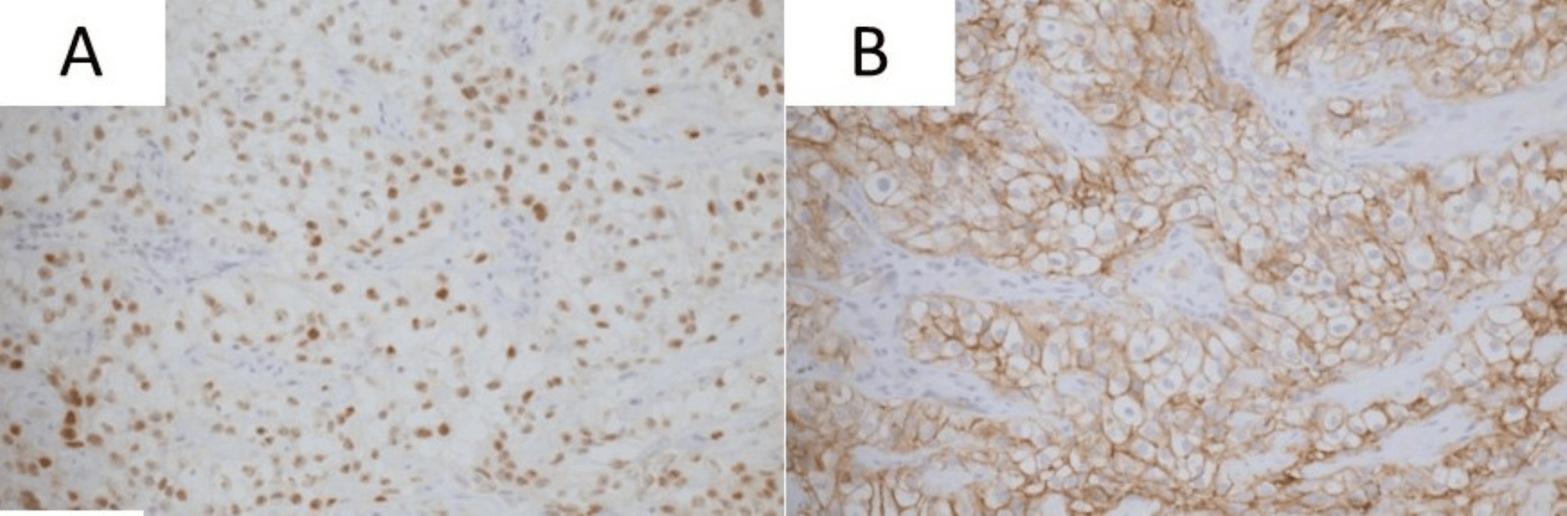
Immunohistochemical staining showing positivity for PAX8 (Panel A), CA9 (Panel B), supporting the diagnosis of metastatic renal cell carcinoma

Immunohistochemistry showed positivity for PAX8, CA9, and CD10 and negativity for CK7, TTF-1, and CD68, confirming metastatic RCC (Figure 5).

The patient was treated for COPD exacerbation with ampicillin/sulbactam and prednisolone, resulting in gradual respiratory improvement. In April 2023, bronchoscopic intervention was performed under fluoroscopic guidance with respiratory surgery backup. A flexible bronchoscope (Olympus BF type 1T260) was used under local anesthesia following endotracheal intubation.

Based on clinical, radiological, and pathological findings, the lesion was diagnosed as an EM of RCC, 12 years after nephrectomy. The patient was referred to a cancer center for systemic therapy with immune checkpoint inhibitors and targeted agents.

## Discussion

EM is a rare form of intrathoracic dissemination from extrapulmonary solid tumors, reported in approximately 3.6-8.6% of metastatic cases [1]. RCC is known for its potential to recur long after nephrectomy, with pulmonary metastases occurring in 30-50% of cases [2]. RCC is also considered the most common primary tumor associated with EM [3].

Typical symptoms of EM include cough, dyspnea, and hemoptysis; however, asymptomatic cases have also been reported [4,5]. In this case, differentiation from hilar-type lung cancer was necessary due to the patient’s long smoking history, elevated tumor markers, and imaging findings. Bronchoscopic examination revealed a pedunculated polypoid lesion arising from the membranous portion of the right main bronchus. MCT allowed for safe excision and adequate tissue sampling, enabling a definitive diagnosis.

One of the primary minimally invasive procedures, Nd:YAG laser cauterization, carries a risk of airway ignition during hypoxemia when administered under high-concentration oxygen. On the other hand, MCT reduces the risk of ignition because thermal energy is generated by the high vibration of water molecules. Two years later, recurrence was observed at the same site in this case, and MCT was performed. The prognosis for EM from RCC is generally poor, with survival typically ranging from one to two years.

Interventional procedures using flexible bronchoscopy carry the risk of airway obstruction, and securing the airway is essential. Collaboration with thoracic surgeons and preparation for percutaneous cardiopulmonary support (PCPS) are crucial for safe execution. In this case, preoperative coordination with respiratory surgery and PCPS ensured procedural safety.

Rigid bronchoscopy offers additional therapeutic options, including airway dilation and emergency management of obstruction [6]. EM can be classified into polypoid lesions protruding into the lumen and infiltrative lesions causing luminal narrowing [7]. This case was consistent with the polypoid type.

Kiryu et al. described four potential pathways for EM: (1) direct metastasis to the bronchus, (2) invasion from pulmonary metastases, (3) infiltration from metastatic lymph nodes, and (4) progression from peripheral lung metastases into the airway [8]. In this case, mediastinal lymphadenopathy suggested both hematogenous and lymphatic spread.

Late recurrence of RCC, defined as recurrence more than 10 years after nephrectomy, occurs in 3.7-11.0% of cases [9-11]. The median interval to EM onset after RCC surgery has been reported as 82 months [4,12]. Recent genomic studies have revealed subtype-specific oncogenic mechanisms in RCC, suggesting the possibility of both early and late recurrence [13].

## Conclusions

This case illustrates the potential role of MCT in treating rare tracheal metastases originating from RCC. In situations where surgical resection is not feasible due to anatomical complexity or patient comorbidities, MCT may offer a safe and effective alternative that preserves surrounding structures and minimizes procedural risk. The absence of major complications and the favorable local response underscore the importance of individualized, minimally invasive strategies in managing complex oncologic presentations. Further accumulation of similar cases may help refine procedural protocols and clarify patient selection criteria, ultimately contributing to broader clinical acceptance of MCT in rare metastatic scenarios. As this is a single case report, the efficacy of MCT for EM in RCC requires verification in a larger, long-term, multipatient study.
